# Variation in emotion dynamics over time is associated with future relationship outcomes

**DOI:** 10.3389/fnhum.2024.1331859

**Published:** 2024-03-28

**Authors:** Simran K. Johal, Emilio Ferrer

**Affiliations:** Department of Psychology, University of California, Davis, Davis, CA, United States

**Keywords:** dyadic interactions, emotion dynamics, dynamic modeling, generalized additive modeling, vector autoregressive model, non-stationarity, romantic relationships

## Abstract

Romantic relationships are defined by emotion dynamics, or how the emotions of one partner at a single timepoint can affect their own emotions and the emotions of their partner at the next timepoint. Previous research has shown that the level of these emotion dynamics plays a role in determining the state and quality of the relationship. However, this research has not examined whether the estimated emotion dynamics change over time, and how the change in these dynamics might relate to relationship outcomes, despite changes in dynamics being likely to occur. We examined whether the magnitude of variation in emotion dynamics over time was associated with relationship outcomes in a sample of 148 couples. Time-varying vector autoregressive models were used to estimate the emotion dynamics for each couple, and the average and standard deviation of the dynamics over time was related to relationship quality and relationship dissolution 1–2 years later. Our results demonstrate that certain autoregressive and cross-lagged parameters do show significant variation over time, and that this variation is associated with relationship outcomes. Overall, this study demonstrates the importance of accounting for change in emotion dynamics over time, and the relevance of this change to the prediction of future outcomes.

## Variation in emotion dynamics over time is associated with future relationship outcomes

Romantic relationships can be defined by the emotional interdependence of the two people involved (Kelley et al., 2002; Vallacher et al., 2005). Indeed, relationships can be viewed as “temporal interpersonal emotions systems,” such that the emotion state of one partner at one timepoint influences how the other partner feels at the same or future timepoint (Butler, 2011; Lougheed and Hollenstein, 2018). Although this interdependence of emotional states has been given different names (e.g., synchrony, reciprocity, transmission, contagion, coregulation, and coupling), we will use the term *emotion dynamics* to refer to the influence that one partner's emotions have on either their own emotions or their partner's across time. Over time, these moment-to-moment emotion dynamics can reveal important aspects of the couple, such as the quality of their relationship (Granic, 2005; Lougheed and Hollenstein, 2018).

Much work has examined how emotion dynamics relate to important outcomes in romantic relationships (Gottman and Levenson, 1992; Gottman, 1994; Gottman et al., 1998; Saxbe and Repetti, 2010; Castro-Schilo and Ferrer, 2013; Sels et al., 2016; Gonzales et al., 2018). However, little work has been done to understand how change or variation in these dynamics over time can also contribute to such outcomes, despite the expectation that such change could or should occur. The rest of the introduction focuses on reviewing the relation between emotion dynamics and relationship outcomes. We then discuss why it is important to consider variation in emotion dynamics and outline the goals of the current study.

### Association of emotion dynamics with relationship outcomes

Numerous studies have shown that emotion dynamics exist within many romantic relationships, and that these dynamics are associated with the state of the relationship. Emotions and stresses of one member of a couple can influence the emotions and stresses of their partner, and the level of this influence can vary depending on several factors, such as characteristics of the couple, the context in which the couple is interacting, or the particular emotions being studied (Bolger et al., 1989; Ferrer and Nesselroade, 2003; Butner et al., 2007; Schoebi, 2008; Saxbe and Repetti, 2010; Randall and Butler, 2013; Randall and Schoebi, 2015; Sels et al., 2016).

These emotion dynamics can relate to the state of the relationship, such as how satisfied the couple feels or whether they remain together, with some of the most notable research in this area having been done by Gottman and colleagues. They found that greater exchange of negative emotions between a marital couple during a conversation (as evidenced by couples engaging in conflict, displaying negative behaviors such as being critical of their partner, or reciprocating their partner's negative behaviors) was related to the risk of marital dissolution and marital satisfaction (Gottman and Levenson, 1992; Gottman, 1994; Gottman et al., 1998). Along similar lines, couples where the wives' negative affect was more strongly related to their husbands' negative affect were less likely to be satisfied with the marriage (Saxbe and Repetti, 2010).

The association between emotion dynamics and relationship outcomes is not limited to just the influence between negative affect states, however. Ferrer et al. (2012) found that emotion synchrony of a couple, conceptualized as when the two partners reported being in similar affective states, predicted whether the couple remained together 1 or 2 years later. Castro-Schilo and Ferrer (2013) found that dynamic parameters describing the interactions between each partner's positive and negative affect were predictive of relationship quality, but not relationship dissolution, above and beyond their level of affect. Gonzales et al. (2018) found that dynamic parameters relating the female partner's affect to her male partner's affect were predictive of relationship dissolution. Finally, Sels et al. (2016) found that the wellbeing of a couple's relationship is associated with how emotionally interdependent the couple is.

### Variation in emotion dynamics

Almost all work focused on emotion dynamics in romantic couples has assumed that such dynamics are constant over time. In other words, the influence that one partner's emotions have on their partner's emotions remain the same over the course of the relationship. Yet we might expect the emotion interdependence of a couple to not remain constant, and instead change over time.

For example, emotion convergence states that a couple's emotions should become more similar over time. This increasing similarity, or increasing covariation, of the couple's emotions is thought to be beneficial because it helps the couple better respond to the demands of the environment and feel close to each other (Anderson et al., 2003; Vallacher et al., 2005; Butler, 2011; Sels et al., 2018). This increasing emotion similarity might occur due to the influence of one person's emotions on those same emotions of their partner becoming stronger over time, reflecting a change in the relationship's emotion dynamics.

The idea that emotion dynamics should change over time is further supported by empirical research. Thompson and Bolger (1999) found that emotion dynamics can change due to the presence of a stressful event: as one partner approached the date of a stressful exam, the influence of their negative mood on their partner's feelings declined. This reduction in interdependence was thought to be due to the receiving partner making more allowances for their partner's negative mood. But changes in emotion interactions do not need to be marked by an external event, and can simply occur over the course of time or due to internal processes (such as ruminating on an experience or the experience of particular affective states; De Haan-Rietdijk et al., 2016; Bringmann et al., 2018).

Although it is realistic to expect changes in emotion dynamics amongst members of a couple, no research has investigated whether the presence or magnitude of these changes relate to relationship outcomes in the same way that the (mean) level of the emotion dynamics do. Previous research focusing on the emotions of one partner have shown that greater variation in one partner's evaluations of the relationship (e.g., their level of satisfaction, how committed they are to the relationship, how committed they perceive their partner to be) is related to relationship instability or greater displays of negative behavior toward their partner (Kelley, 1983; Arriaga, 2001; Arriaga et al., 2006; Campbell et al., 2010). In these situations, greater variability in relationship evaluations is believed to reflect problems in the relationship, resulting in negative outcomes for the couple. Yet this research has only focused on variability in one partner's feelings toward the relationship without considering interdependence in emotions at all. And although there has been work showing the benefits of increased emotion similarity on relationships (Anderson et al., 2003; Townsend et al., 2014), these studies did not explicitly measure variation in emotion dynamics.

To understand why it is important to think about variation in emotion dynamics when studying relationship outcomes, consider the following example. Suppose we had measured the positive and negative emotions of a husband and wife over time. At the beginning of the study, the effect of the husband's negative mood is negatively related to his wife's negative mood, such that she downplays her own negative mood in an attempt to make allowances for his. Yet, over time, this dynamic wears on her, and the husband's negative mood begins to have a stronger effect such that there is a contagion in negative mood from him to her. If the escalation continues, the increasingly negative interactions between the couple result in the end of their relationship, as Levenson and Gottman (1985) and Gottman et al. (1998) have shown that negative affect reciprocity is the strongest indicator of an unhappy marriage and low marital satisfaction. If we had not measured the change in emotion dynamics, then we would have estimated the effect of the husband's negative mood on his wife's negative mood as non-existent or weak (due to the change from a strong negative effect to a strong positive effect over time). Thus, in this hypothetical example, measuring emotion dynamics alone was not enough to help us learn why the couple ended their relationship. Instead, it was necessary to measure the change in emotion dynamics, as the presence of that change contained important information related to the state of the relationship.

### The present study

Although understanding how levels of emotion dynamics relate to relationship outcomes is important, accounting for and understanding changes in emotion dynamics over time is equally important. This helps researchers not only accurately characterize the process under study, but could also reveal useful information that is not available from standard, time-invariant parameters. If the dynamics in a relationship change over time due to factors indicating relationship stress (e.g., one member of a couple becoming less emotionally receptive, or external events such as one partner losing their job), then being able to measure such changes in the parameters could be useful for predicting the future of the relationship. The aim of the present study is to examine whether the change in emotion dynamics over time, as measured by time-varying vector autoregressive models, is related to relationship outcomes above and beyond the mean of those dynamics alone. In the following sections, we describe the data used to answer our questions, and the time-varying vector autoregressive model used to measure variation in emotion dynamics over time. We then investigate whether these changes are predictive of relationship outcomes—in particular, perceived relationship quality and relationship dissolution—and conclude with a discussion of the future directions and limitations of our approach.

## Methods

### Participants

Data were collected as part of the Dynamics of Dyadic Interactions Project (DDIP), a longitudinal study examining the emotion dynamics of couples over time (Ferrer et al., 2012). The two members of each couple were asked to complete a daily diary questionnaire for up to 90 days, with questions pertaining to their general emotional affect and their affect with respect to their relationship. In order to have enough data to estimate the TV-VAR models, and to remain consistent with previous work (Castro-Schilo and Ferrer, 2013), we limited our analyses to couples who had completed at least 50 days of the questionnaire. We further limited our analyses to those couples who had provided follow-up information on their relationship 1 or 2 years later. This process resulted in a total of 148 couples. Participants in this subsample ranged in age from 17 to 74 years (*M* = 24.22, *SD* = 9.34), and had been in a relationship from 1 month to 35.1 years (*M* = 2.93 years, *SD* = 5.40). Of the 148 couples, 28 reported living together while 120 reported not living together. Additionally, 2 of the couples reported their relationship status as “dating around,” 99 reported dating each other exclusively, 8 reported being engaged, and 26 were married. The remaining 13 couples reported living together but did not report their relationship status. On average, participants provided 65.5 days of data (*SD* = 15.7), although each dyad was missing, on average, ~3.96% of their total daily diary data (*SD* = 9.32%).

### Measures

#### Relationship affect

Relationship-specific affect (RSA; Ferrer and Widaman, 2008; Ferrer et al., 2012) is a questionnaire designed to measure affect related to one's relationship. The questionnaire consists of 18 items intended to capture both positive (nine items) and negative affect (nine items). The instructions read, “Indicate to what extent you have felt this way about your relationship today.” Participants rated all items using a Likert-type scale ranging from 1 (*very slightly or not at all*) to 5 (*extremely*). Previous work with this scale has demonstrated good psychometric properties regarding reliability of change within person (Cranford et al., 2006), indicating the precision of the measurement of systematic change of persons across days, with reliability coefficients for positive and negative affect of 0.85 and 0.87 (for females) and 0.82 and 0.85 (for males; Ferrer et al., 2012).

#### Relationship outcomes

One and two years after the initial visit, participants returned for a set of follow-up interviews. As part of these interviews, participants were asked about their relationship status, and were recorded as having broken up if they were no longer with their initial partner at either of the two follow-up interviews. Participants were also asked about their relationship quality, which was assessed using six items from the Perceived Relationship Quality Component Inventory (Fletcher et al., 2000). These items included questions such as “How satisfied are you with your relationship?” and “How committed are you with your relationship?” and were answered on a 7-point Likert scale (1 = *Not at all* and 7 = *Extremely*). The scores for each member of a couple were then averaged together to form one overall relationship quality score.

### Statistical models to examine emotion interactions

#### Vector autoregressive (VAR) models

One common statistical approach to model multivariate time series is the vector autoregressive (VAR) model. In the VAR model, each variable being studied (in our case, the positive and negative affect of the female partner and the positive and negative affect of the male partner toward the relationship) is predicted by itself and all other variables at previous timepoints up to a certain lag (with the most common being a lag-1 VAR model, denoted as VAR(1), in which each variable is predicted by itself and all other variables at the previous timepoint only). In other words,


$$\begin{array}{l} {\text{y}}_{t}=\text{c}+\text{Φ}{\text{y}}_{t-1}+\;{\text{ϵ}}_{t} \end{array}$$

In this model, **c** contains the intercepts of the model, **ϵ**_*t*_ contains the model residuals, or whatever part of the observed process was not explained by the variables at the previous timepoints, and **Φ** is a matrix that contains the autoregressive parameters on the diagonal and cross-lagged parameters on the off-diagonal (Shumway and Stoffer, 2011). The autoregressive parameters represent the effect of a variable's own value at the previous timepoint on itself at the current timepoint, controlling for the effect of all other variables. In the context of the variables studied here, an example autoregressive effect would be how the female partner's positive affect toward the relationship relates to her positive affect toward the relationship the next day. The autoregressive effect is commonly interpreted as the stability of the process or inertia—a higher positive autoregressive effect indicates that the process takes longer to revert to equilibrium, or that the individual is more “rigid” in that state. For example, if the female partner was feeling very favorable to her relationship at one timepoint (high positive affect), then a positive autoregressive effect indicates that she would likely continue to feel favorable toward her relationship (have high positive affect) at the next timepoint. On the other hand, a negative autoregressive effect indicates a more rapidly fluctuating or oscillating process, as a high value at one timepoint would typically be followed by a lower value at the next timepoint. So, if the female partner in our example felt very favorable about her relationship at one timepoint, then she would likely feel less favorable to her relationship (low positive affect) the next day, which is then followed by feeling very favorable again the day after.

The cross-lagged parameters represent the effect of one variable under study (e.g., positive affect of the female partner) at the previous timepoint on a different variable (e.g., negative affect of the female partner) at the current timepoint, controlling for all other autoregressive and cross-lagged effects. A positive cross-lagged effect indicates that a high value on one process at a particular timepoint would generally lead to a high value on the other process at the next timepoint, and in the context of emotion dynamics, could represent emotion amplification or emotion escalation (Sbarra and Ferrer, 2006; Sels et al., 2016). A negative cross-lagged effect indicates that a high value on one process at a particular timepoint generally leads to a low value on the other process at the next timepoint, and could represent emotion reversal or emotion dampening (Sbarra and Ferrer, 2006; Sels et al., 2016). For example, suppose that our cross-lagged parameter of interest represented the influence the female partner's negative affect toward the relationship yesterday had on her male partner's positive affect toward the relationship today. A positive value of this cross-lagged parameter would mean that if the female partner felt very negative toward the relationship yesterday, then the male partner is likely to feel more positive to the relationship today. A negative value, on the other hand, would mean that if the female partner felt very negative toward the relationship yesterday, then the male partner is likely to not feel very positive (low positive affect) toward the relationship today.

The above example also demonstrates that the cross-lagged parameters contain the effects between different affect states within the same partner (e.g., female partner's positive affect to her own negative affect), the effects between same affect states across partners (e.g., female partner's negative affect to her male partner's negative affect), and the effects between different affect states across partners (e.g., female partner's negative affect to her male partner's positive affect). The former parameters (different affect states, same partner) can be referred to as intra-partner effects, while the latter sets of parameters (same affect states across partners and different affect states across partners) can be referred to as inter-partner effects.

Since the autoregressive and cross-lagged parameters encode the associations between the variables at the previous timepoint and the variables at the current timepoint, we will refer to them as dynamic parameters in the rest of the paper. This is because these parameters represent the emotion dynamics of the couple by quantifying the influence emotion states have on each other within and across partners.

The VAR model specified above assumes that the processes under study are all stationary, or that the mean, variance, and covariances of the processes all remain constant over time (Shumway and Stoffer, 2011). Stationarity further implies that the model parameters are constant over time, which, as mentioned above, is unlikely to hold when studying emotion interactions over any relatively long period. Therefore, it is preferable to use a model that either does not assume stationarity or is able to account for departures from this assumption, in order to more accurately characterize the process under study and not obtain biased estimates of the dynamic parameters (Ryan et al., 2023).

#### Time-varying VAR model

One way to account for non-stationarity in psychological processes is to allow the VAR model parameters to vary over time. This model, called the time-varying VAR (TV-VAR) model, allows any combination of the intercepts, autoregressive effects, and cross-lagged effects to take on different values at each timepoint (Bringmann et al., 2017, 2018):


$$\begin{array}{l} {\text{y}}_{t}={\text{c}}_{t}+{\text{Φ}}_{t}{\text{y}}_{t-1}+{\text{ϵ}}_{t} \end{array}$$

Although the TV-VAR model relaxes the stationarity assumption of the VAR model, it still requires that the process is stationary at any given timepoint, and that the model parameters change in a gradual, as opposed to abrupt, fashion.

Multiple methods, such as kernel-smoothing and regression splines, are available to estimate these time-varying parameters (Bringmann et al., 2017, 2018; Haslbeck et al., 2021). In this paper, we use the generalized additive modeling (GAM) framework, which uses penalized thin-plate regression splines to estimate how the coefficients change over time. One benefit of this approach is that *how* the time-varying parameter changes over time does not need to be specified in advance but is instead estimated from the data. More specifically, each time-varying coefficient (here, the intercept terms, autoregressive effects, and cross-lagged effects) is written as a function of *k* known basis functions *R(t)*:


$$\begin{array}{l} \phi_{ijt}={\hat{\alpha }}_{1}R_{1}\left(t\right)+{\hat{\alpha }}_{2}R_{2}\left(t\right)+\ldots +{\hat{\alpha }}_{k}R_{k}\left(t\;\right) \end{array}$$

Each added basis function determines how flexible or “wiggly” the final functional form of the time-varying coefficient is, with each basis function being “wigglier” than the previous. At the same time, the estimated regression coefficients ($\hat{\alpha_{1}},\hat{\alpha_{2}},\ldots ,\hat{\alpha_{k}}$) control how much weight that basis function is given at each timepoint. To prevent the functional form from being too flexible, the regression coefficients are estimated using a penalized likelihood approach, so that the effect of the more flexible basis functions are downplayed due to a “wiggliness penalty.” The optimal penalty is estimated using a generalized cross-validation technique, so that the final form is neither too wiggly (the penalty is too low) or too smooth (the penalty is too high). Readers interested in more details about this method are referred to Bringmann et al. (2018) and Haslbeck et al. (2021).

### Data analysis

**Figure 1:**
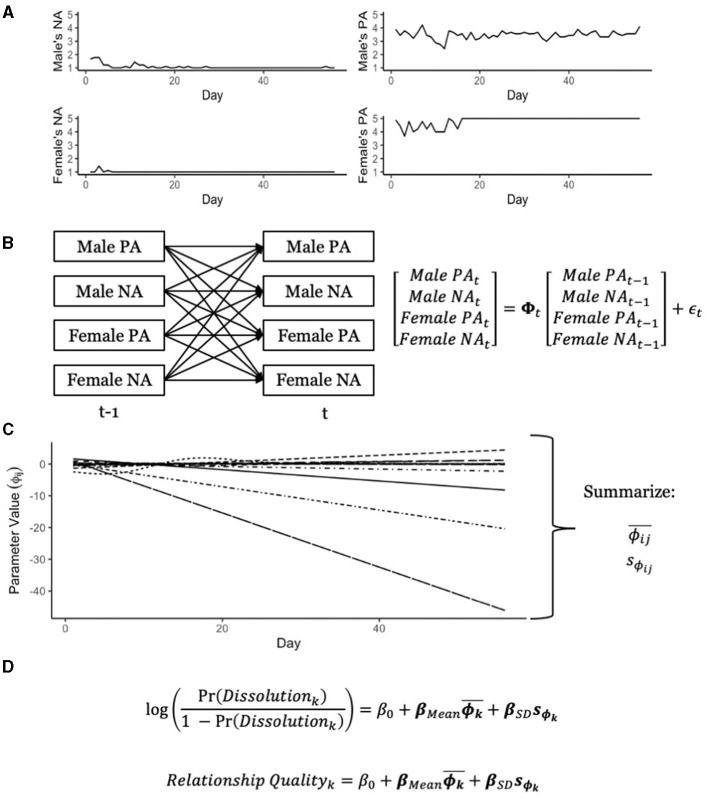
Description of data analysis procedure. **(A)** Displays an example time series of positive and negative affect for each member of a couple. **(B)** Shows the vector autoregressive model used to analyze this data, such that the autoregressive and cross-lagged effects are allowed to change over time. **(C)** Shows how the autoregressive and cross-lagged effects change over time, and these effects are each summarized into a mean and standard deviation. Finally, in **(D)**, the means and standard deviations of the dynamic parameters are used to predict relationship dissolution and relationship quality.

We were interested in examining whether incorporating information on the variation of emotion dynamics over time was associated with future relationship outcomes, even after controlling for the mean value of those parameters. The data analysis procedure to answer this question is displayed in Figure 1 and described in more detail below.

As mentioned above, each dyad rated to what extent they felt certain positive or negative emotions toward their relationship that day, and the ratings were averaged into a positive affect (PA) and negative affect (NA) score for each member of the couple (Figure 1A). This resulted in four time series (PA of the male partner, NA of the male partner, PA of the female partner, and NA of the female partner), which were then used to estimate a separate TV-VAR model for each dyad (Figure 1B). These analyses were conducted in R (R Core Team, 2022, version 4.2.1), using the package *mgcv* (Wood, 2017). The number of basis functions was kept at 10 (the default in *mgcv*), and inspection of the results showed that this was sufficient. In other words, the effective degrees of freedom for all smooth functions were not close to 10, indicating no more basis functions needed to be added. Finally, *mgcv* handled missing data by using listwise deletion, such that days where at least one variable was missing information was not used in model estimation.

The output of interest from the TV-VAR model was the estimate of **Φ**_*t*_ at each timepoint for each dyad that, as displayed in Figure 1C, could potentially show substantial variation over time. To summarize each dyad's estimated **Φ**_*t*_ matrices in a way that could then be used as predictors of the external outcomes, we calculated the mean and standard deviation for each dynamic parameter over time. Thus, the final estimates obtained from fitting the TV-VAR model to each dyad were 16 means and 16 standard deviations of the corresponding dynamic parameters.

In the final step, the means and standard deviations of the dynamic parameters were used as predictors of relationship quality and relationship dissolution in a linear and a logistic regression model, respectively. Significant associations were chosen based on a stepwise regression approach, with both forwards and backwards selection. Although there are noted drawbacks to stepwise regression (Steyerberg et al., 1999; Austin and Tu, 2004), we chose to proceed with this approach due to the relatively high number of predictors (16 means and 16 standard deviations of dynamic parameters, 32 total), compared to the number of dyads (127 dyads).

## Results

### Descriptive statistics

Of the 148 couples (and thus, the 148 TV-VAR models), 127 successfully converged, with the remaining 21 failing to converge due to an insufficient number of timepoints with complete data. Descriptive statistics across these couples for the positive and negative affect variables (averaged across time), the outcome variables, and covariates are presented in Table 1, while descriptive statistics for the means and standard deviations of the dynamic parameters over time are presented in Table 2. A visualization of the change in dynamic parameters for each dyad is available at: https://github.com/skjohal/Dyadic-Affect-Networks. By the 2-year follow-up, 29 of the 127 couples (or 22.83%) had ended their relationship, and most couples reported high levels of relationship quality (*M* = 5.96, *SD* = 0.77).

**Table 1:** Descriptive statistics for raw positive and negative affect, outcome variables, and covariates of interest.

	**Male PA**	**Male NA**	**Female PA**	**Female NA**	**Total involvement**	**Initial rel. quality**	**Final rel. quality**	**Rel. Dissolution**
Mean	3.54	1.35	3.50	1.34	3.09	6.26	5.96	0.23
SD	0.66	0.31	0.68	0.28	5.74	0.61	0.77	-
Maximum	1.53	1.01	1.65	1.00	0.04	3.09	3.58	-
Minimum	4.93	2.70	4.83	2.37	35.08	7.00	7.00	-
Male PA^a^	1							
Male NA	−0.31	1						
Female PA	0.76	−0.31	1					
Female NA	−0.31	0.61	−0.42	1				
Total involvement	0.01	−0.18	0.00	−0.16	1			
Initial rel. quality	0.42	−0.24	0.50	−0.36	0.08	1		
Final rel. quality	0.43	−0.20	0.46	−0.21	0.02	0.46	1	
Rel. dissolution^b^	−0.29	0.19	−0.29	0.33	−0.63	−0.32	−0.42	1

PA, Positive Affect; NA, Negative Affect; Rel., Relationship.

a PA and NA for each member of a couple were first averaged over time. These descriptive statistics are then across dyads, not across time.

b Since relationship dissolution is a binary variable (1 = the couple has ended their relationship), the mean reflects the percentage of couples who have ended their relationship in our sample, and there is no SD, minimum, or maximum value. Furthermore, correlations with this variable are point-biserial correlations.

**Table 2:** Descriptive statistics for mean and variability of dynamic parameters over time.

**Variables**	**Mean**	**SD**	**Min**	**Max**
**Mean of dynamic parameters**				
Female PA → Male PA	0.06	0.22	−0.76	0.73
Female NA → Male PA	0.01	0.46	−3.17	1.24
Male PA → Male PA	0.05	0.25	−0.60	0.75
Male NA → Male PA	0.08	0.50	−1.31	2.61
Female PA → Male NA	−0.01	0.24	−0.64	1.09
Female NA → Male NA	−0.13	2.05	−22.7	2.20
Male PA → Male NA	−0.02	0.20	−1.01	0.58
Male NA → Male NA	0.001	0.27	−0.76	0.65
Female PA → Female PA	0.06	0.30	−0.81	1.53
Female NA → Female PA	−0.07	0.51	−3.29	1.72
Male PA → Female PA	0.06	0.28	−0.61	1.48
Male NA → Female PA	0.07	0.54	−1.21	2.81
Female PA → Female NA	−0.02	0.21	−0.69	0.92
Female NA → Female NA	−0.06	0.95	−10.27	0.89
Male PA → Female NA	−0.03	0.24	−1.18	0.89
Male NA → Female NA	0.03	0.31	−0.96	1.01
**Variability of dynamic parameters**				
Female PA → Male PA	0.17	0.13	< 0.01	0.53
Female NA → Male PA	0.30	0.39	< 0.01	3.66
Male PA → Male PA	0.19	0.18	< 0.01	1.03
Male NA → Male PA	0.39	0.44	< 0.01	2.07
Female PA → Male NA	0.14	0.16	< 0.01	0.82
Female NA → Male NA	0.36	1.27	< 0.01	13.9
Male PA → Male NA	0.15	0.17	< 0.01	1.1
Male NA → Male NA	0.23	0.18	0.02	0.82
Female PA → Female PA	0.18	0.20	0.01	1.70
Female NA → Female PA	0.38	0.43	< 0.01	2.91
Male PA → Female PA	0.24	0.23	< 0.01	1.53
Male NA → Female PA	0.47	0.78	< 0.01	7.93
Female PA → Female NA	0.15	0.13	< 0.01	0.56
Female NA → Female NA	0.25	0.54	< 0.01	5.99
Male PA → Female NA	0.19	0.21	< 0.01	1.21
Male NA → Female NA	0.34	0.42	< 0.01	2.63

PA, Positive Affect; NA, Negative Affect.

Mean of dynamic parameters refers to the mean of each autoregressive and cross–lagged effect for each dyad over time, whereas variability of dynamic parameters is the standard deviation of each effect for each dyad over time. The descriptive statistics (mean, SD, min, and max) are then calculated on these means and SDs across dyads.

To determine whether a parameter was time-varying or time-invariant, we evaluated whether the smooth function was statistically significant and the effective degrees of freedom were >2, as these are indications that the smooth function is: (1) important to the model and (2) non-linear (Bringmann et al., 2017, 2018). Based on these criteria, few dynamic parameters showed variation over time. On average, two out of the 16 dynamic parameters were significantly time-varying. However, this varied widely across dyads: the number of dynamic parameters showing variation ranged from 0 to 10 for any given dyad. The autoregressive parameters showed more variation over time than the cross-lagged parameters, although cross-lagged parameters did occasionally vary over time. In particular, cross-lagged parameters involving the partner's negative affect at the previous timepoint, such as the relation from both partners' negative affect at the previous timepoint to the female partner's positive and negative affect, and the male partner's positive and negative affect, showed significant variation over time.

### Relation to relationship quality

To examine the association between the mean and variation of the dynamic parameters over time on the relationship quality, we used a stepwise linear regression. The results of the final model are shown in Table 3. With regards to the average of the dynamic parameters over time, only the cross-lagged effect from the female partner's positive affect to her own negative affect was a significant predictor of relationship quality. The greater this cross-lagged effect, the higher the reported relationship quality (*b* = 1.00, *p* = 0.01).

**Table 3:** Summary of model predicting relationship quality.

	***b***	***SE***	***t***	***p***
Intercept	5.97	0.13	46.09	< 0.001
**Means**				
Male PA → Male PA	−0.49	0.30	−1.62	0.11
Female PA → Female NA	1.00	0.35	2.86	0.01
Male NA → Female NA	−0.34	0.22	−1.56	0.12
Female PA → Male PA	0.49	0.33	1.51	0.13
**Variation**				
Female PA → Male PA	−1.33	0.57	−2.34	0.02
Female PA → Male NA	0.64	0.47	1.38	0.17
Male NA → Female NA	0.43	0.16	2.62	0.01

PA, Positive Affect; NA, Negative Affect.

Then, looking at the variation of the dynamic parameters over time, change in the cross-lagged effects from the female partner's positive affect to the male partner's positive affect, and from the male partner's negative affect to the female partner's negative affect, were both significant predictors. The greater the variation in the cross-lagged effect from the female partner's positive affect to her male partner's positive affect, the lower the relationship quality (*b* = −1.33, *p* = 0.02). However, greater variation in the effect from the male partner's negative affect to the female partner's negative affect was related to higher relationship quality (*b* = 0.43, *p* = 0.01).

Although the means and standard deviations of some dynamic parameters predicted relationship quality, the model only explained 9.12% of the total variation. However, the regression model performed better than an intercept-only model, Δχ^2^(7) = 2.81, *p* = 0.01, indicating the relative contribution of the predictors included in the model.

### Relation to relationship dissolution

To examine the relation between the dynamic parameters from the time-series model and relationship dissolution we used a stepwise logistic regression with both forward and backward entry, using the means and standard deviations of the dynamic parameters across time as predictors. Table 4 includes the results from the final model, which included a total of eight predictors, with six of them significant.

**Table 4:** Summary of model predicting relationship dissolution.

	***b***	***SE***	***t***	***p***	**Odds ratio**
Intercept	−2.16	0.47	−4.59	< 0.001	0.22
**Means**					
Male NA → Male NA	−2.98	1.23	−2.42	0.02	0.05
Female PA → Female PA	−2.36	1.23	−1.92	0.06	0.09
Female PA → Female NA	−3.97	1.56	−2.55	0.01	0.02
Male PA → Female NA	−1.77	1.26	−1.40	0.16	0.17
Male NA → Female NA	3.66	1.15	3.18	0.00	39.0
**Variation**					
Female NA → Male PA	2.01	0.87	2.30	0.02	7.43
Male NA → Male PA	−2.09	0.96	−2.18	0.03	0.12
Male PA → Female NA	3.19	1.22	2.62	0.01	24.3

PA, Positive Affect; NA, Negative Affect.

Looking first at the means of the dynamic parameters over time, three were significant: the average autoregressive effect of the male partner's negative affect, the average cross-lagged effect from the female partner's positive affect to her own negative affect, and the average cross-lagged effect from the male partner's negative affect to the female partner's negative affect. The effects of the stability of the male partner's negative affect, and the cross-lagged effect from the female partner's positive affect to her negative affect, on relationship dissolution were both negative (Male NA → Male NA: *b* = −2.98, *p* = 0.02, odds = 0.05; Female PA → Female NA: *b* = −3.97, *p* = 0.01, odds = 0.02), indicating that stronger influence between these affect states decreased the likelihood of breaking up. On the other hand, the cross-lagged effect from the male partner's negative affect to the female partner's negative affect was positive (*b* = 3.66, *p* = 0.001, odds = 38.86), such that stronger cross-lagged coefficients greatly increased the chances of the couple breaking up.

Furthermore, variation over time in the dynamic parameters involving the male partner's positive affect were also predictive of relationship dissolution. Variation in the cross-lagged effect from the female partner's negative affect to the male partner's positive affect, the cross-lagged effect from the male partner's negative affect to his own positive affect, and the cross-lagged effect from the male partner's positive affect to the female partner's negative affect were significant predictors. The cross-lagged effects between the male partner's positive affect and the female partner's negative affect were both positive (Female NA → Male PA: *b* = 2.01, *p* = 0.02, odds = 7.43; Male PA → Female NA: *b* = 3.19, *p* = 0.008, odds = 24.28), indicating that greater variation in these effects over time increased the chances of the couple ending their relationship. However, the effect from the male partner's negative affect to his own positive affect was negative (*b* = −2.09, *p* = 0.03, odds = 0.12), such that greater variation in this cross-lagged effect over time decreased the chances of relationship dissolution.

The classification accuracy of the final logistic regression model was 81.1%, which was an improvement from the 77.2% accuracy of the intercept-only model. Furthermore, the logistic regression model showed improved fit relative to the intercept-only model, with Cox and Snell *R*^2^ = 0.24 and Nagelkerke *R*^2^ = 0.36.

### Controlling for time in relationship and initial relationship quality

Due to the likelihood that the probability of a couple ending their relationship and their perceived relationship quality are associated with the length of time the couple has been together and their initial relationship quality, we conducted separate analyses controlling for these two variables. In these models, we kept the same predictors that had been identified as important in the initial analysis, and additionally controlled for the time the couple had been together as well as the perceived relationship quality at their initial visit. The results of these models predicting relationship quality and relationship dissolution are shown in Tables 5, 6, respectively.

**Table 5:** Summary of model predicting relationship quality, controlling for time in relationship and initial quality.

	***b***	***SE***	***t***	***p***
Intercept	2.81	0.69	4.07	< 0.001
**Means**				
Male PA → Male PA	−0.39	0.30	−1.32	0.19
Female PA → Female NA	0.83	0.33	2.51	0.01
Male NA → Female NA	−0.20	0.21	−0.97	0.33
Female PA → Male PA	0.45	0.30	1.49	0.14
**Variation**				
Female PA → Male PA	−1.24	0.56	−2.21	0.03
Female PA → Male NA	0.72	0.44	1.61	0.11
Male NA → Female NA	0.30	0.15	1.91	0.06
**Control variables**				
Time in relationship	−0.00	0.01	−0.03	0.97
Initial relationship quality	0.51	0.11	4.69	< 0.001

PA, Positive Affect; NA, Negative Affect.

**Table 6:** Summary of model predicting relationship dissolution, controlling for time in relationship and initial quality.

	***b***	***SE***	***t***	***p***	**Odds ratio**
Intercept	6.22	3.07	2.03	0.04	502.59
**Means**					
Male NA → Male NA	−2.76	1.33	−2.08	0.04	0.06
Female PA → Female PA	−1.63	1.34	−1.22	0.22	0.20
Female PA → Female NA	−3.66	1.73	−2.12	0.03	0.03
Male PA → Female NA	−2.38	1.41	−1.68	0.09	0.09
Male NA → Female NA	3.44	1.25	2.76	0.01	31.22
**Variation**					
Female NA → Male PA	1.55	0.89	1.74	0.08	4.69
Male NA → Male PA	−1.35	0.96	−1.42	0.16	0.26
Male PA → Female NA	3.60	1.42	2.54	0.01	36.51
**Control variables**					
Time in relationship	−0.22	0.16	−1.37	0.17	0.81
Initial relationship quality	1.31	0.51	−2.57	0.01	0.27

PA, Positive Affect; NA, Negative Affect.

When predicting relationship quality, accounting for initial relationship quality and length of time in the relationship removed many of the effects of emotion dynamics. The only predictors related to emotion dynamics that remained significant were the average cross-lagged effect from the female partner's positive affect to her own negative affect (*b* = 0.83, *p* = 0.01) and the amount of variation in the cross-lagged effect from the female partner's positive affect to the male partner's positive affect (*b* = −1.24, *p* = 0.03). The effects were in the same direction as before: A greater average value of the effect from the female partner's positive affect to her own negative affect was related to greater relationship quality, while greater variation in the effect from the female partner's positive affect to the male partner's positive affect was related to decreased relationship quality, even after controlling for length of time in the relationship and initial relationship quality. As expected, initial relationship quality was also a significant predictor of future relationship quality, such that those with higher initial relationship quality tended to have higher relationship quality at the follow-up interviews (*b* = 0.51, *p* < 0.001).

Initial relationship quality was also related to the probability of ending the relationship, such that couples with higher initial relationship quality had a lower chance of ending their relationship (*b* = −1.31. *p* = 0.01, odds = 0.27). Emotion dynamics continued to be related to relationship dissolution: all emotion dynamics averaged over time that were significant in the initial model remained significant, and one parameter related to variation in emotion dynamics remained significant. Greater average values of the male partner's autoregressive effect for negative affect and the cross-lagged effect from female partner's positive affect to her own negative affect were related to decreased risk of relationship dissolution (Male NA → Male NA: *b* = −2.76, *p* = 0.04, odds = 0.06; Female PA → Female NA: *b* = −3.66, *p* = 0.03, odds = 0.03). Furthermore, a greater average value in the cross-lagged effect from the male partner's negative affect to the female partner's negative affect, and greater variation in the cross-lagged effect from male partner's positive affect to female partner's negative affect, was related to increased risk of ending the relationship (Male NA → Female NA: *b* = 3.44, *p* = 0.01, odds = 31.22; Male PA → Female NA: *b* = 3.60, *p* = 0.01, odds = 36.51).

Overall, initial relationship quality was a significant predictor of both future relationship quality and relationship dissolution. However, even after controlling for this and length of time in the relationship, variation in emotion dynamics continued to play a role.

## Discussion

The aim of our present work was to examine whether changes in emotion dynamics of a couple over time was associated with future relationship outcomes, to help us better understand the role that emotion dynamics can play within a relationship. To answer this question, we estimated TV-VAR models and calculated means and standard deviations of the autoregressive and cross-lagged parameters across time. Our TV-VAR models indicated that there was variation over time in these dynamic parameters for each dyad, although this variation tended to be small. Typically, only two out of the 16 dynamic parameters showed significant variation over time for each dyad. The parameters that tended to vary the most were the autoregressive effects, although this may be due to the general tendency for autoregressive effects to be stronger and, thus, more likely to be significant than cross-lagged effects. Since one of our criteria for evaluating whether a parameter was significantly time-varying was whether its smooth function was statistically significant (i.e., the parameter plays an important role in the model), this may have resulted in more autoregressive parameters being detected as significantly time-varying.

Some findings about which parameters tend to be significantly time-varying also align with previous research. For example, negative emotions tend to be more contagious (have more significant influence) than positive emotions (Larson and Almeida, 1999). We extended this finding by showing that parameters representing the influence of negative affect on other affect states are not only more contagious, but they also vary over time more frequently than parameters representing the influence of positive affect.

In terms of relations with future outcomes, the results of our stepwise regressions showed that the average of particular autoregressive and cross-lagged effects, as well as variation in other dynamic parameters, was related to both relationship quality and relationship dissolution. Even after controlling for time spent in the relationship and initial relationship quality, variation in the parameters representing emotion dynamics was related to both these outcomes.

Putting these results in the context of other research on the same dataset, we find some similarities in our results pertaining to the average value of the dynamic parameters and those of Castro-Schilo and Ferrer (2013), despite the differences in modeling strategies. Both found that the mean cross-lagged effect from the female partner's positive affect to her own negative affect was a significant, positive predictor of relationship quality. That is, the stronger the influence of the female partner's positive affect on her negative affect the next day, the higher the couple rated the quality of their relationship. However, unlike Castro-Schilo and Ferrer (2013), who found that dynamic parameters did not significantly contribute to the prediction of relationship dissolution, we found that a handful of autoregressive and cross-lagged effects were significantly—and typically negatively—related to relationship dissolution.

It is a little harder to directly compare our results relating relationship dissolution to the average value of the dynamic parameters to the results of Gonzales et al. (2018), due to the use of composites in our analysis and individual items in theirs. However, there was general agreement in the type of dynamic parameters that were significantly related to relationship dissolution. In both our analyses, the female partner's influence on her male partner's affect was a better predictor than the male partner's influence on his female partner's affect, which contrasts with some previous findings showing that husbands' affect has a stronger effect on marriage quality than wives' affect (Cowan and Cowan, 2000). Furthermore, Gonzales et al. (2018) restricted their analysis to only inter-partner effects (from one partner to another), whereas our analysis included intra-partner effects (from one partner's affect to their own affect) and showed the relevance of autoregressive effects to relationship dissolution. However, whereas the results of Gonzales et al. (2018) highlighted the potentially protective nature of the female partner's positive affect—e.g., cross-lagged effects involving indicators of the female partner's positive affect was related to decreased risk of relationship dissolution—our results showed differently. In other words, most of the significant cross-lagged effects involved the female partner's negative affect, and these cross-lagged effects were related to a decreased risk of relationship dissolution.

Our findings help us better understand emotion dynamics between members of a romantic couple, and the role they play in determining relationship outcomes, by explicitly studying how *changes* in emotion dynamics over time can impact relationship outcomes. Changes in the dynamic parameters over time can be potentially indicative of important changes in the relationship, such as increased relationship stress, which then relate (negatively) to future relationship outcomes. Some of our findings align with this interpretation, as well as the general findings of Arriaga (2001) and Arriaga et al. (2006), such that greater variation in the cross-lagged effect from the female partner's positive affect to the male partner's positive affect and in the bidirectional effect between the male partner's positive affect and the female partner's negative affect predicted decreased relationship quality and increased risk of relationship dissolution, respectively.

However, it is important to note that variation in dynamic parameters was not always related to negative relationship outcomes. For example, variation over time in the cross-lagged effect from the male partner's negative affect to his partner's negative affect was linked to *increased* relationship quality. Similarly, greater variation in the cross-lagged effect from the male partner's negative affect to his own positive affect was linked to *decreased* risk of relationship dissolution. Although these effects are counter-intuitive to an interpretation where greater variation is linked to greater stress in the relationship, we believe that they *can* align with an interpretation where greater variation is caused by a change in the dynamic parameter from a value that represents maladjustment in the relationship to a value that represents a healthier relationship state. This would align, for example, with research relating emotion convergence and emotion similarity to positive relationship outcomes (Anderson et al., 2003; Townsend et al., 2014).

For example, we mentioned in the Introduction work demonstrating that couples whose negative emotions are coupled together tend to be less satisfied and more likely to end their marriage (Gottman and Levenson, 1992; Gottman, 1994; Gottman et al., 1998). Although greater variation in the influence that, for example, a female partner's negative affect has on her male partner's affect could mean that this relation goes from being strongly negative to strongly positive, it could also go from being strongly positive to being weak or negative. A strongly positive effect would mean that high values of the female partner's negative affect toward the relationship lead to higher values of the male partner's negative affect, resulting in reciprocation of negative emotions. A weak or negative effect, on the other hand, would mean the female partner having negative feelings toward the relationship does not affect her partner's negative feelings, or leads him to have a decrease in negative feelings to compensate. Therefore, a change from a positive influence to weak or negative influence would represent a healthier state of the relationship. Greater variation in this particular dynamic could be linked to higher relationship quality, as it moves the couple away from a dynamic that tends to reflect maladjustment.

### Limitations and future directions

Despite our results showing the importance of variation in emotional interactions for predicting relationship outcomes, it is important to note that the improvement in model fit compared to an intercept-only model was relatively low for both models. For example, the *R*^2^ value for both models was below 0.40, and the change in classification accuracy for the logistic regression model was only 3.9%. However, this could be explained by several reasons. First, for the logistic regression model, only around 23% of the couples had ended their relationship by the time of the follow-up, and this low base rate could hinder the predictive ability of the model. Secondly, our model treated the dyad as a closed system, such that there were no external inputs or variables other than the mean and variation of the dynamic parameters that related to the outcomes of interest. However, research has shown that there are a variety of socio-demographic factors that could contribute to relationship quality and relationship dissolution, which may not affect the average value or variation of the dynamic parameters (Conger et al., 1990; Gottman and Levenson, 1992; Lewin, 2005; Poortman, 2005; Røsand et al., 2014; Hensel and O'Sulliban, 2022). Finally, and more generally, there was a 1-to-2-year gap between the assessment of daily affect, and the follow-up with assessment of relationship outcomes. Therefore, although change in emotion dynamics could be a potential indicator for important changes in a relationship, it is possible that the effect of variation in emotional dynamics (or the underlying causes of that change) is not as important 1–2 years later as it would have been if the follow-up assessment had occurred, say, 3 months after the daily diary portion of the study. Despite these limitations, there was still enough of an association between the mean dynamic parameters and their variation over time to be picked up in our analysis, and even after controlling for relevant covariates such as length of the relationship and initial perceived quality.

Furthermore, the relation between variation in dynamic parameters and any outcome is reliant on there *being* variation in the dynamic parameters. Although our results did indicate some variation in the dynamic parameters, and that this variation was related to relationship outcomes, very few dynamic parameters showed significant amounts of variation over time. Although this could indicate that emotional dynamics in our sample showed little change over time, it is also possible that we simply did not have enough data to observe any variation that was present. Dyads in our sample mostly provided between 50 and 60 days, and previous work has shown that this number of timepoints might not always be sufficient for the TV-VAR model to detect changes in dynamics (Bringmann et al., 2018). Another possibility is that changes in emotion dynamics are more likely to occur for certain types of couples than others (e.g., those who have just begun their relationship, or those who are close to significant events such as engagement or marriage). Therefore, a future direction of this work could be to repeat this analysis with a larger dataset that includes more timepoints or with a group of couples at similar stages in their relationship, to determine the generalizability of these results beyond this sample.

Future research could also extend the work presented here to gain an even deeper understanding of changes in emotion dynamics and their relation to future outcomes. For example, we mentioned that greater variation in emotion dynamics could reflect a change toward a healthier dynamic, or a change toward a worse dynamic. Yet since we quantified variation using the standard deviation, our measure—although simple—is not able to characterize *how* emotion dynamics change over time, and how the direction of this change is related to relationship outcomes. Therefore, one potential future direction is to replicate these analyses with more fine-grained measures of variation that could give us this information. Furthermore, we mentioned that external events could spark changes in emotion dynamics as well as potentially impacting the relationship directly. Therefore, an interesting future direction would be to examine whether incorporating information about external events helps us understand changes in emotion dynamics [although this might require a model that allows for explicit change points in the dynamic parameters, e.g., Albers and Bringmann (2020)], as well as aids our prediction of relationship outcomes.

## Conclusion

In conclusion, our paper underscores the importance of identifying whether and how couples' exchange of affect varies over time. Given that emotion dynamics unfold over time, capturing such dynamics properly requires using models that can estimate not only the dynamics themselves, but also their variation over time. By using such a model, we revealed variation in dynamic parameters that otherwise would have been ignored. In addition, we showed how such variation was related to future outcomes, such as relationship status and quality, above and beyond the means of such parameters.

## Data availability statement

The data analyzed in this study is subject to the following licenses/restrictions: the use of the dataset can be discussed with EF. Requests to access these datasets should be directed to EF, eferrer@ucdavis.edu.

## Ethics statement

The studies involving humans were approved by University of California, Davis IRB. The studies were conducted in accordance with the local legislation and institutional requirements. The participants provided their written informed consent to participate in this study.

## Author contributions

SJ: Conceptualization, Formal analysis, Writing—original draft. EF: Data curation, Writing—review & editing.
